# Idiopathic Organ Transplant Chorioretinopathy after Liver Transplantation

**DOI:** 10.1155/2015/964603

**Published:** 2015-03-16

**Authors:** Maria Fernanda Abalem, Pedro Carlos Carricondo, Sergio Luis Gianotti Pimentel, Walter Yukihiko Takahashi

**Affiliations:** Retina and Vitreous Department, Universidade de São Paulo, Brazil

## Abstract

Idiopathic organ transplant chorioretinopathy is a rare disease associated with kidney and heart transplantation. We present a case secondary to liver transplantation including its multimodal imaging, differential diagnosis, and physiopathology discussion.

## 1. Introduction

Central serous chorioretinopathy (CSC) in the setting of solid organ transplantation (SOT), especially kidney, is a well-described condition. Although still poorly understood, it seems to be multifactorial, probably related to the use of corticosteroids and immunosuppressive agents, underlying disease and type of organ transplant received. Four distinct patterns have been described, based on fluorescein angiography (FA), including diffuse retinal pigment epitheliopathy (DRPE), focal CSC, multifocal CSC, and CSC with bullous retinal detachment (RD) [1, 2].

Gass et al. described four cases of posterior idiopathic chorioretinopathy, another and broader spectrum of clinical findings, in patients after kidney and heart-lung transplantation. The similarity among them was the presence of geographic zones of disruption and coarse clumping of the retina pigment epithelium (RPE), associated to RD [3].

We describe a case of idiopathic organ transplant chorioretinopathy (IOTC) following liver transplantation, assessed by FA, indocyanine green angiography (ICG), fundus autofluorescence (FAF), retinal optical coherence tomography (OCT), and enhanced depth imaging by spectral-domain OCT (EDI).

## 2. Case Report

A 60-year-old man was referred to the Ophthalmology Department for assessment of progressive blurred vision in both eyes, in the past two years. He had a liver transplantation 3 years before, due to liver failure after hepatitis C. He developed chronic arterial hypertension and mild renal insufficiency as collateral effects of transplant medication. There were no medical records of transplant rejection. The medication in use was acetylsalicylic acid (100 mg), anlodipine (5 mg), furosemide (40 mg), tacrolimus (1 mg), and mycophenolate mofetil (360 mg), all for three years. He also had a past history of corticosteroid use (5 to 10 mg) for 6 months after the transplant.

**Figure 1:**
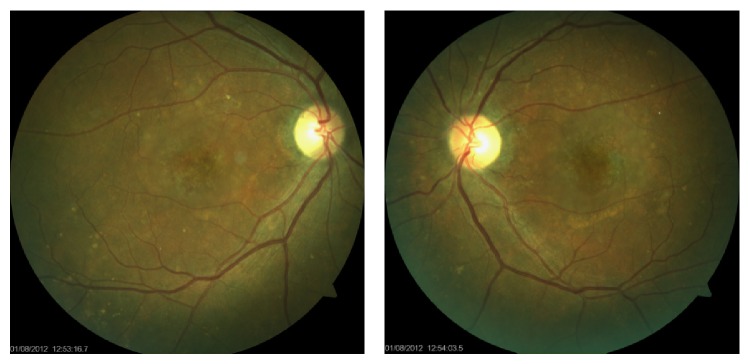
Ocular fundus.

**Figure 2:**
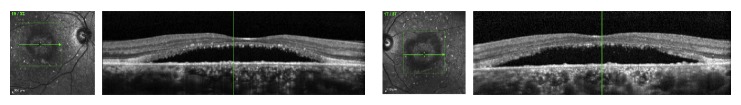
Spectral-domain OCT.

**Figure 3:**
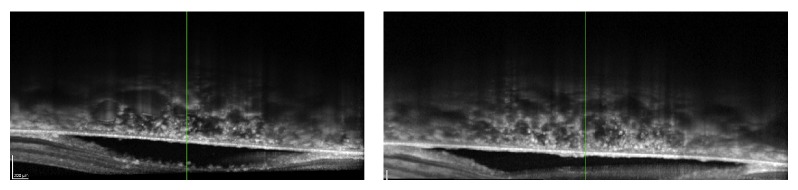
EDI.

**Figure 4:**
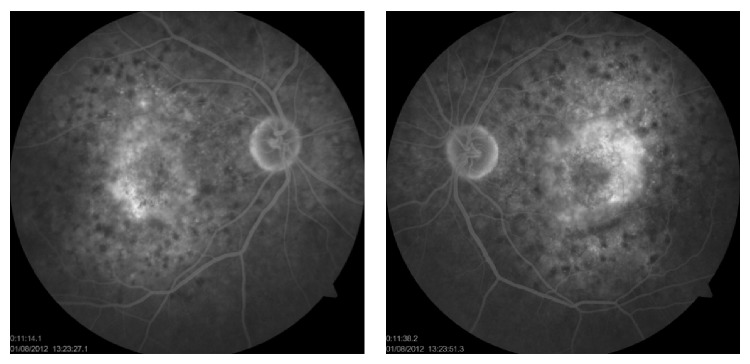
Fluorescein angiography.

**Figure 5:**
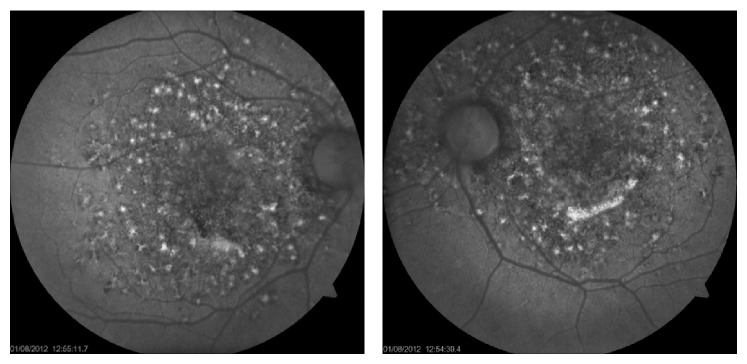
Fundus autofluorescence.

**Figure 6:**
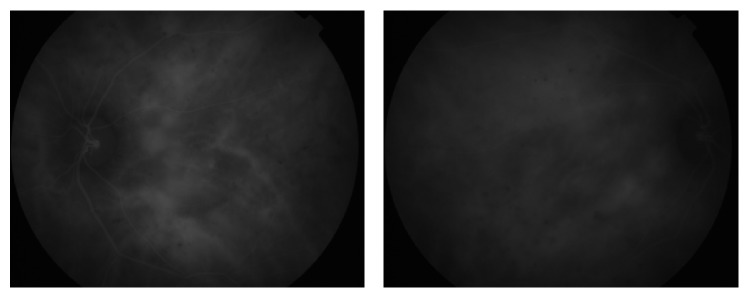
ICG.

In the ophthalmological examination, he presented a corrected visual acuity of 20/200 in both eyes. There was no pupillary reflex defect. The biomicroscopy was unremarkable. The fundoscopy (Figure 1) showed mild hypertensive retinopathy and mottled pigmentary changes in posterior pole. The Spectralis SD-OCT (Heildelberg Engineering, Heildelberg, Germany) evidenced a serous macular detachment with protein-enriched fluid, without RPE detachment (Figure 2). The EDI evidenced a thickened choroid (450 *μ*, measured subfoveal, from the outer border of RPE to the inner border sclera) with hyperreflective dots (Figure 3). FA (Visucam 500, Zeiss, Germany) showed a distinct pattern of multiple small (100 *μ*), round hypofluorescent dots, and a diffuse hyperfluorescent fundus due to pooling, especially around the fovea (Figure 4). The FAF (Visucam 500, Zeiss, Germany) showed round granular hyperautofluorescence lesions corresponding to the hypofluorescent areas on the FA, with a granular hypoautofluorescent fundus in posterior pole (Figure 5). The ICG (TRC 50-X retinal camera and Imaginet 50X Imaginet Systems, Topcon Inc., Tokyo, Japan) presented with multifocal areas of choroidal vascular hyperpermeability, without focal leakage (Figure 6).

Although our patient did not show prominent RPE changes in the fundus examination, the FA findings were similar to the “leopard skin” pattern. We could also observe dye pooling around the fovea consistent with serous macular detachment, without specific points of leakage. This fact is important in order to distinguish this condition from idiopathic chronic CSC or CSC after solid organ transplantation, since IOTC does not present with focal leakage [4]. Moreover, it is also distinct from DRPE, characterized by widespread distribution of small pigment epithelial detachments (PEDs), a few RPE changes, and the presence of more extensive pigmentary alterations with variable leakage on the FA [5].

The use of FAF among several retinal conditions has been well established [6]. This patient presented with granular and confluent hyperautofluorescent lesions, associated with granular hypoautofluorescent areas. Additionally, hypofluorescent spots on the FA were correlated with hyperautofluorescence areas on FAF, showing a RPE distress, with lipofuscin deposition. These mottled RPE changes were not fully observed with ocular fundi. It suggests that FAF might better assess early RPE changes with the additional advantage of being a noninvasive tool, considering that patients in the setting of kidney or liver transplantation may have contraindication to dye injection. Spaide and Klancnik Jr. reported diverse findings of FAF in CSC. In chronic stages, hyperautofluorescent lesions with material seemed to gravitate inferiorly or to collect in deposits in the border of the detachment. In the absence of subretinal fluid, decreased autofluorescence lesions were seen, associated with geographic atrophy and fluid descending tracts [7]. Our patient showed a very distinct pattern, suggesting that the FAF may play a role in the differential diagnosis of IOTC.

The ICG is an adjunct diagnosis tool to assess diseases affecting the choroid. In this case, the ICG presented with multifocal areas of choroidal vascular hyperpermeability, similar to CSC [8]. These similar ICG findings arise questions about similarity in pathogenesis with CSC.

The advance of OCT provided a better understanding of serous detachments, especially those located in the macular region. Our patient presented with a serous macular detachment, rich in protein exudation (hyperreflective dots) and no pigment epithelial detachments (PED). In a recent study about CSC after renal transplantation (RT), the OCT images revealed subretinal fluid and multiple small PEDs [9]. Patients with organ transplant are indeed subject to neurosensory macular detachment or CSC probably due to corticosteroid use. However, patients with IOTC present with serous detachment with the particularity of absence of PEDs [1]. Thus, besides the pigmentary changes, the pattern of macular detachment is distinct from idiopathic and isolated CSC after solid organ transplant.

Another aspect was the presence of a thickened subfoveal choroid (575 *μ*) associated with hyperreflective points, assessed by EDI. The average normal choroidal thickness is 275–300 *μ*, although a correlation of choroidal thickness with age, axial length, and refraction has been found. Increasing age and myopia are associated with choroidal thinning [10]. Choroidal thickening in patients with CSC has been reported in the literature [11]. Jirarattanasopa et al. also recently described choroidal thickening in CSC subtypes, in agreement with vascular hyperpermeability seen in ICG [12]. Considering that IOTC and CSC may have similar pathogenesis with regard to choroidal function, the EDI may be an alternative and noninvasive tool to evaluate the choroid in these patients.

## 3. Discussion

We described a patient who presented with bilateral idiopathic chorioretinopathy after liver transplantation. Gass et al. have reported this entity in 1992, following kidney and heart-lung transplantation. The common features of this condition are zones of mottled disruption of RPE and yellow pigment clumping in the macular area in both eyes, which appear hypofluorescent at the FA (“leopard skin” appearance). Also, a serous RD may be present as a hyperfluorescent area on FA [3]. This is the first case in literature with these characteristics secondary to liver transplantation.

The cause of segmental areas of RPE derangement with serous RD after solid organ transplantation is still unknown. In fact, it was only analyzed after kidney and heart transplants. Diverse elements have been suggested in the pathogenesis of this condition, including use of corticosteroids or/and immunosuppressive agents; underlying cause leading to the transplant; presence of systemic hypertension; and graft rejectio [3]. In our case, the patient was not under corticosteroids use and only used it for 3 months after transplantation (5–10 mg/day).

Immunosuppressive agents are largely used for diverse systemic and ocular diseases. In this manner, a larger incidence of this condition would be expected in patients under these drugs. Moreover, there are no reports about these drugs causing any other secondary RD [3].

Hepatitis C virus infection has been associated with many ocular disorders such as ischemic retinopathy, dry eye, scleritis, and peripheral ulcerative keratitis [13]. Nevertheless, in the four cases reported by Gass et al., there were any patients with hepatitis C infection; therefore virus infection is not the probable cause of IOTC [3]. The use of Pegylated interferon, commonly prescribed in hepatitis C, has been implicated in vasocclusive ocular complications [14]. However, the patient has not used it.

Systemic hypertension has been associated with CSC in patients with organ transplantation, but not especially in patients with IOTC [15]. In fact, IOCT resembles retinopathies with a hypertensive background, like accelerated idiopathic hypertension and severe toxemia of pregnancy [3]. Nevertheless, despite the secondary systemic hypertension in this patient, it was well controlled with antihypertensive agents and signs of hypertensive retinopathy were not important.

With regard to graft rejection, three types of mechanisms are described: hyperacute, acute, and chronic. In hyperacute rejection, preformed antibodies react with alloantigens on the vascular endothelium, activate complement, and trigger intravascular thrombosis and necrosis of the vessel wall. In acute rejection, CD8^+^ T lymphocytes reactive with alloantigens on graft endothelial cells and parenchymal cells damaging these cell types. Finally, in chronic rejection, T cells react with graft alloantigens and produce cytokines that induce proliferation of endothelial cells and intimal smooth muscle cells, causing luminal occlusion [16]. All mechanisms are common to all organ transplants and lead to vascular injury. It could have happened in the choroidal vessels, leading to an ischemic process, similar to severe hypertensive states, which may explain the retinopathy seen in all cases. To our knowledge, although our patient did not have any record of graft rejection, it is possible that fundus changes may represent a similar mechanism to the systemic organ rejection, involving choroid and retina tissues.

Until now, we can only hypothesize about IOTC pathogenesis. Further studies and histopathologic data are necessary to explain the chorioretinal complications described in this report.
